# Supplementary material for: Intrinsic functional architecture of the non-human primate spinal cord derived from fMRI and electrophysiology
Source: Nat Commun. 2019 Mar 29;10:1416. doi: 10.1038/s41467-019-09485-3 (PMC6440970; doi:10.1038/s41467-019-09485-3)
Supplement: Supplementary file 1 — Supplementary Information [file 41467_2019_9485_MOESM1_ESM.pdf]

Intrinsic functional architecture of the non-human primate spinal cord derived from fMRI and electrophysiology

Wu et al.

SUPPLEMENTARY INFORMATION

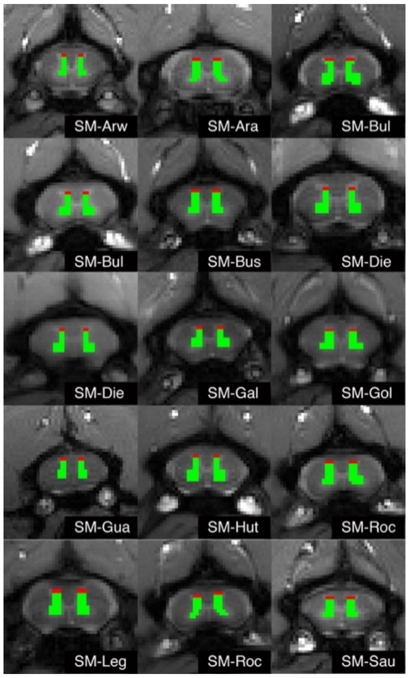

Supplementary Figure 1: ROIs drawn for each monkey to generate correlation values in Figure 4C. A total of 15 imaging sessions were used to scan 12 animals, and hence repeated monkey labels are shown. Red and green voxels represent dorsal seed regions and different depth voxels respectively.

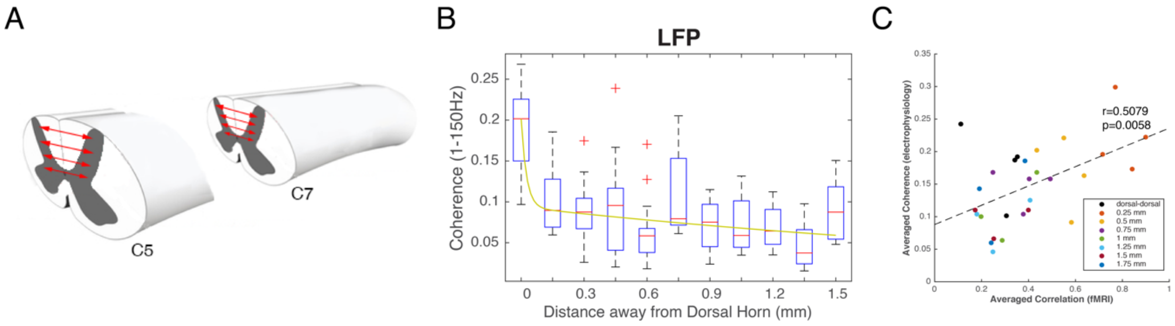

Supplementary Figure 2: (A) Schematic diagram of coherences computed in the recordings of the spinal cord. Coherences between contralateral horns as a function of penetration depth were computed along the dorsal horn to the intermediate-gray-matter section. (B) Within-slice connectivity patterns across the spinal cord as a function of laminar depth for LFP. Because penetration depths of each monkey are slightly different, there are 18, 15, 11 and 8 observations for each boxplot between 0-0.60mm, 0.75-1.05mm, 1.20-1.35mm and 1.50mm respectively. (C) Linear regression of electrophysiology coherence versus rsfMRI correlation for the four monkeys that underwent electrophysiology. Coherence values at each depth were computed relative to its dorsal horn voxel seed; similar to what was performed for fMRI. Coherence values were interpolated linearly to find coherences at corresponding depths in MRI. Coherences and correlations were averaged between runs (and shanks) to produce one data point for each monkey at each depth. Correlation between the two modalities were computed to be 0.5079 (p=0.0058).

Supplementary Table 1: P-values of two-sided Mann-Whitney statistical comparison tests between groups of connectivity values at various depths relative to the I-GM from Figure 4C

	Voxels away from intermediate GM								
	-5	-4	-3	-2	-1	0	1	2	3
p-value (uncorrected)	1.67×10^{-10}	1.27×10^{-11}	4.62×10^{-11}	2.68×10^{-7}	2.70×10^{-2}	N/A	7.55×10^{-1}	8.14×10^{-2}	2.53×10^{-2}
p-values (FDR-corrected)	5.02×10^{-10}	1.14×10^{-10}	2.08×10^{-10}	6.02×10^{-7}	4.05×10^{-2}	N/A	7.55×10^{-1}	9.15×10^{-2}	4.05×10^{-2}
p-values (Bonferroni corrected)	1.34×10^{-9}	1.02×10^{-10}	3.69×10^{-10}	2.14×10^{-6}	2.16×10^{-1}	N/A	6.04×10^{0}	6.51×10^{-1}	2.02×10^{-1}

Supplementary Table 2: P-values of Pearson's r-values uncorrected and corrected from Figure 4C

	Voxels away from intermediate GM								
	-5	-4	-3	-2	-1	0	1	2	3
p-value (uncorrected)	0	1.96×10^{-7}	1.51×10^{-5}	2.05×10^{-4}	3.59×10^{-4}	1.43×10^{-2}	1.61×10^{-2}	9.07×10^{-3}	2.82×10^{-5}
p-values (Bonferroni corrected)	0	1.70×10^{-5}	1.31×10^{-3}	1.78×10^{-2}	3.12×10^{-2}	1.15×10^{0}	1.39×10^{0}	7.36×10^{-1}	2.45×10^{-3}
